# Does the bank-firm human relationship still matter for SMEs? The game-changing role of digitalization

**DOI:** 10.1007/s11187-023-00758-2

**Published:** 2023-04-04

**Authors:** Francesco Fasano, Tiziana La Rocca

**Affiliations:** 1 Department of Business Administration and Law, University of Calabria, 87036 Rende, CS Italy; 2 Department of Economics, University of Messina, 98122 Messina, Italy

**Keywords:** Management, SMEs, Bank debt, Digitalization, Banking relationship, Information asymmetries, G30

## Abstract

Noteworthy contributions have highlighted that human contact is a considerable factor in bank-firm relationships. It allows the acquisition of soft information, which alleviates information asymmetries and increases the use of bank debt. The advent of digital technologies in the information collection process open new horizons and change the role of personal contacts in bank-firm interactions, as entrepreneurs visit bank branches less frequently. This study uses a large sample of Italian SMEs from 2011 to 2020 and finds that the rapid increase and use of digital instruments have reduced the positive influence of physical closeness between banks and SMEs on the indebtedness levels. Interestingly, our study has also found that the COVID-19 crisis did not amplify this moderating effect. Results support theories that human contact is an important factor in bank-firm relationships because it allows the acquisition of soft information, which alleviates information asymmetries and increases the use of bank debt. Our study suggests that close human ties are still extremely relevant and digitalization should be exploited to support the collection of the kind of qualitative soft information that is crucial in debt negotiations.

## Abstract (plain language summary)

The growing integration and digitalization of banks gives rise to a question: are local personal bank-entrepreneur relationships still important for small businesses? We interestingly find that human relationships between the bank and the firm occurring at the local level are important in the soft information collection procedures, positively influencing the amount of debt that SMEs use. In light of such relevance, governments’ challenge consists on directing banks toward a system that strengthens the collection of soft information in a digitalized world. In a phygital future, digitalization should thus be exploited to provide banks not only quantitative data, but also qualitative information that support lending decisions.

## Introduction

The future of banking will be influenced by the rapid development of digitization, which has revolutionized the financial services industry over the years (Puschmann, 2017). The use of new technologies has changed the banking business worldwide, and “financial technology” (fintech^1^) that is based on digitalization has become essential in the banking relationship^2^ (Romānova & Kudinska, 2016). During the last few years, banks have increased their investment in IT, which accounts for 15–20% of their total costs (Gopalan et al., 2012). Digitalization has become a challenge but also an opportunity, as it provides more flexibility, better functionality, and the aggregation of banking services (Romānova & Kudinska, 2016). These recent tendencies have stimulated a growing academic interest in this field, generating a rapid increase in the number of papers studying the relationship between banks and digitalization. A recent paper by Thakor (2020) reviewed the existing literature on new technologies and its interaction with banking. The author points out that there is still much that “we do not know” in this literature context. Among the many factors involved in the bank-fintech relationship, the role that digitalization plays in the information collection procedure of banks is worthy of attention (Jakšič & Marinc, 2019). Credit contracts are almost exclusively based on information (Puschmann, 2017), and the information gathering process was historically based on repeated human contacts between a firm and its bank branch (Diamond, 1984). The mitigation of bank-firm information asymmetries^3^ is the essence of the banking relationship (Greenbaum et al., 2016), and it bases its foundations on the human interactions that allow the bank to acquire soft information^4^ about companies, thereby facilitating loan provision. Indeed, face-to-face meetings between bankers and entrepreneurs simplify screening and monitoring activities, reducing the information gap. This is particularly important for informationally opaque firms, typically small- and medium-sized enterprises (SMEs) that have limited access to external finance because of their asymmetric information problems (Beck et al., 2005; Berger & Udell, 1998).

The extant literature suggests that the availability of soft information increases when the proximity between lenders and entrepreneurs is greater (Agarwal & Hauswald, 2010). In particular, some studies have underscored that bank branch concentration favours human relationships and the collection of non-quantifiable soft information that is difficult to obtain in impersonal ways (Liberti & Petersen, 2019; Petersen, 2004) such as digitalization. The noteworthy contribution of Guiso et al. (2004) points out that the density of bank branches is positively related to the corporate growth of SMEs. This work has been enormously influential and has inspired a large body of literature. For instance, La Rocca et al. (2010) found that bank-firm physical closeness alleviated asymmetric information problems and increased SMEs’ use of bank debt. This work suggests that banking relationship is stronger when human contacts are more frequent. Hence, SMEs benefit from the proximity of loan officers, who can rapidly assess their credit worthiness (Alessandrini et al., 2009; Deloof & La Rocca, 2015; Deloof et al., 2019; La Rocca et al., 2010).

In this context, where information is of extraordinary value, the advent of new digital technologies in the information collection process has opened up a completely new frontier that could revolutionize the role of human contact in bank-firm relationships in the coming years. Digitization could play a breakthrough role in this context, as it is an interesting new opportunity to improve the way banks collect information about their customers. The use of digital platforms has evolved mightily over the years (Acs et al., 2021). Web-based banking platforms, which allow to carry on banking operations without geographical limitations (Khedmatgozar & Shahnazi, 2018), provide big data^5^ (Cappa et al., 2020a, 2020b) that empowers a bank’s capacity to collect standardized information. Big data play a key role in this process. Indeed, generating, collecting, and analysing data is nowadays easy and fast thanks to information technology. Consequently, collection procedures of hard information do not require the physical presence of the entrepreneur. Thus, in such a way big data could significantly affect the banking relationship. Digitalization provides advanced automation of the information gathering process thanks to the Internet. As shown by Jakšič and Marinc (2019), this innovation does not mean that a bank should eliminate its close personal interaction with an entrepreneur, but rather that it should take this opportunity to overcome certain weaknesses in its information collection activities. A bank could take advantage of digitalization in order to reduce its “distance” from a firm when it is logistically difficult to have live personal interaction. In this regard, an advanced method of collecting quantifiable hard information based on the artificial intelligence exploited by digitalization could strengthen the bank-firm relationship by integrating and not replacing human ties, which are inevitably characterized by bounded rationality (Jakšič & Marinc, 2019).

Our present work, based on asymmetric information theory, studies whether the explosive increase in digital instruments in the last decade has shaped the role of personal contacts between bankers and entrepreneurs to reduce information asymmetries. In a context in which the use of internet services influences the collection of information (Arnold & van Ewijk, 2011; Blasio, 2009), we aim to address this research question: does the advent of digitalization moderate the effect bank-firm physical closeness has on SME financial policies?

Our results, using a very recent database, highlight the finding that bank-firm physical proximity has a positive effect on SME use of debt. Moreover, they show that digitalization negatively moderates such positive effect. Furthermore, this moderating effect is not shaped by the COVID-19 crisis and does not exist for cooperative bank branches.

Our contribution suggests that, despite the fact digitalization is rapidly spreading around the banking world, human relationships are still extremely important. After less than 20 years from the milestone work of Guiso et al. (2004), we find it interesting that bank branch proximity is still important, even in a world where technology dominates the scene in almost all sectors. SMEs need human bankers, and personal contacts cannot be fully replaced by digitalization. For instance, the discretion of a loan officer can hardly be substituted by digitalization, and this is particularly important for informationally opaque SMEs, whose access to external finance is very important (Finnegan & Kapoor, 2023). Thus, a key implication of our findings is that the importance of bank-firm geographical closeness is changing, which means that in the near future, banking institutions will have to rethink their business models in light of the ongoing growth of digitization. A new idea of bank-firm digital proximity could complement the benefits of physical proximity. In addition, the recent coronavirus pandemic has changed the approach firms have toward banks, as entrepreneurs appreciate online services that are accessible everywhere (Alhassany & Faisal, 2018). This and constant IT expansion should persuade governments to support banks during their online transition in order to strengthen the bank-firm relationship. As soft information still matters even in a digitalized world (Estrin et al., 2022), another important implication of our research is that banks should try to use digital instruments to acquire not only hard, but also soft information (and, when possible, codify it). Consequently, the artificial intelligence techniques that form the basis of digitalization could also support the strategic and qualitative decisions of banks, which would consequently have a strong positive impact on bank-firm relationships.

## Literature review and development of hypotheses

### Literature review

Digitalization, which consists on transforming information into a digital item, is significantly taking place in the banking sector. However, there is no single definition of digitalization in the banking world (Schueffel, 2016), and it is also difficult to quantify (Thakor, 2020), as it is a developing force which refers to a broad set of technological financial innovations (Schueffel, 2016). Indeed, finance and technology meet each other in many respects (e.g. blockchains, crowdfunding, peer-to-peer lending, internet banking, mobile payments, cryptocurrencies, robo-advisories, Insurtech, among others). With regard to the banking industry, digitalization made it possible both to have more digital data available and perform online operations. This has brought many benefits, such as the reduction of transaction costs and fake currency, decrease of human errors, and handling large amounts of cash. At the same time, banks are more vulnerable to cyber-attacks, and bank branches changed their role. Digitalization is especially helpful to increase a bank’s knowledge about new customers (Mishra et al., 2022) and could reduce the need of excessive banking control and supervision that could damage the firm (Ahmed, 2021).

The disruptive arrival of digitalization encouraged many researchers to seek a deeper understanding of the advent of technology in this sector. The work of Thakor (2020) has reviewed the existing literature on digitalization and banking. Navaretti et al. (2018) and Vives (2017) point out that digitalization is changing the business models of banking institutions. More generally, the banking world is wondering whether digitalization can completely substitute banks (Boot, 2017). Recently, Hodula (2021) and Cole et al. (2019) highlighted the finding that the current literature does not provide an unequivocal answer concerning the role of fintech as a complement to or substitute for bank finance.

Some works have noted that new digital procedures generate economies of scale in the processing of banking services (Boot, 2016; Li & Marinč, 2018). These advantages arise from the fact that internet banking makes it possible to implement banking activities without geographic limitations (Khedmatgozar & Shahnazi, 2018). Another important stream of research in this field has investigated how hard, non-codifiable information obtained through digitalization could change the role of loan officers who base their lending decisions on soft information collected via direct personal contacts (Uchida et al., 2012). This soft information is the basis for the discretion of a banker (Cerqueiro et al., 2011), and it can be easily acquired when a bank and a firm are physically close (Liberti & Petersen, 2019). Some studies suggest that personal interactions are still important even in a digital banking world (Ferri & Murro, 2015; Grunert & Norden, 2012; Marinč, 2013) because soft information still matters, especially for SMEs that face more asymmetric information problems (Berger & Udell, 1998). Personal contact between entrepreneurs and their banks is more frequent when bank branches and firms operate closely, and thus, firms benefit from bank-firm proximity (Guiso et al., 2004; Kendall, 2012). Guiso et al. (2004) suggest that bank branch concentration is of great significance for corporate growth, despite the globalization of financial markets. The authors have argued that this only applies to informationally opaque SMEs, whose asymmetric information problems make human ties particularly important in their case (Alessandrini et al., 2009; Beck et al., 2005; Pollard, 2003). A close relationship between an SME and a bank, thanks to physical proximity, reduces the asymmetric information gap (Petersen & Rajan, 2002) and, consequently, reduces financial constraints in lending activities. Proceeding from the contribution of Guiso et al. (2004), the literature has examined the relationship between bank-firm geographic proximity and the corporate financial policies of SMEs. Noteworthy articles find that bank branch closeness positively influences firms’ use of debt (González & González, 2008; La Rocca et al., 2010; Palacín-Sánchez & Di Pietro, 2016; Utrero-González, 2007), cash holdings (Fasano & Deloof, 2021), and trade credit (Deloof & La Rocca, 2015). Alessandrini et al. (2009) carried out a study based on the same context as Guiso et al. (2004) and observed that geographic distance between a firm and a bank reduces the amount of debt used by SMEs. La Rocca et al. (2010) similarly observed that bank branch density favours credit provision to SMEs. The same results were observed in Spain, where, just as in Italy, differences in the level of SME debt depended on differences in bank-firm distance (González & González, 2008; Palacín-Sánchez & Di Pietro, 2016; Utrero-González, 2007). Therefore, close contacts between lenders and entrepreneurs facilitate the acquisition of soft information on SMEs (Howorth & Moro, 2006), reducing information asymmetries and increasing access to bank finance.

In essence, the arrival of internet banking increases the efficiency of the information gathering processes of banks, but it decreases banker-entrepreneur human interaction, because transactions can be carried out at a distance. This distance could influence the collection of soft information based on personal contacts. Moreover, as highlighted by the extant literature, banking consolidation and financial technology reduce credit availability, especially for SMEs (Berger & Frame, 2007; Degryse & Ongena, 2005; Sapienza, 2002). It therefore appears to be imperative to investigate how digitalization influences the effect bank-firm proximity can have on SME financial choices.

### Development of hypotheses

Textbooks commonly define asymmetric information as the situation in which one of two parties is better informed than the other, which has implications for credit markets. For instance, Tirole (2006, page 237) argued that “the issuer may raise less funds or raise funds less often when the capital market has limited access to information about the firm”. Asymmetric information problems arise in the presence of adverse selection and moral hazards. Adverse selection occurs when one party does not know the qualities of its counterpart before the contract is closed. Moral hazards take place after the contract is closed, when one of the two parties cannot acquire enough information about their counterpart.

Information asymmetry due to adverse selection and moral hazard problems could affect cash holdings (Chung et al., 2015) and is a major concern in financial markets (Gan & Riddiough, 2008; Leland & Pyle, 1977; Myers & Majluf, 1984; Nier & Baumann, 2003; Stiglitz & Weiss, 1981). Personal contacts between banks and firms are the building blocks of the banking relationship (Diamond, 1984), and they mitigate information asymmetries (Bayless & Diltz, 1991; Greenbaum et al., 2016; Limpaphayom & Polwitoon, 2004). Repeated personal interactions allow banks to acquire the soft information that is fundamental for credit provision (Boot, 2000), and this is difficult to codify. The existing literature has revealed that close proximity between bank branches and firms increases personal contacts, reduces asymmetric information problems, and has a positive effect on the financial policies of firms, especially SMEs (Deloof & La Rocca, 2015; La Rocca et al., 2010). Therefore, to assess whether bank branches proximity still has a positive effect on SMEs’ use of debt in the latest years, in line with the extant contribution, we test our first hypothesis:

- Hypothesis 1: Bank-firm physical proximity positively affects SME debt

In this context, the new digitalization that has transformed the information collection processes of banks could influence the role of bank branch concentration, as digitalization diminishes information asymmetries (Cappa et al., 2020a, 2020b), because hard information (e.g. balance sheets and collateral guarantees) can be standardized through machine learning techniques. Thus, at present, internet-based banking plays a considerable role in reducing information asymmetries in banking. For these reasons, in recent years, banks have increasingly used hard information for their credit evaluations (Liberti & Petersen, 2019). Online platforms make it possible to provide remote banking services (Khedmatgozar & Shahnazi, 2018) and avoid logistical limitations. The technological services provided by banks directly match the bank to the entrepreneur, providing information about the firm’s credit worthiness and its financial needs.

The huge availability of hard information could also have a double “dark side”, however. First of all, internet banking provides hard, standardized information that is often not sufficient to guarantee loan provisions, especially for SMEs suffering from asymmetric information problems. Secondly, internet banking could reduce face-to-face interactions. In this regard, De Young et al. (2007) argued that the distance between banks and firms makes it considerably more difficult for banks to collect valuable information and it increases the probability of default. Indeed, despite digitalization could provide a piece of soft information (for instance, thanks to the content of notes to the financial statements), it is well known that it mainly provides hard information and reduces the need to go to bank branches (Fasano & Cappa, 2022). In light of this reasoning, one has to wonder about the implications to the growth of hard standard information for the human interactions that take place in the rooms of bank branches and, consequently, what the impact is on the amount of bank debt issued for SMEs. Jakšič and Marinc (2019) raise a question: “Is online and mobile banking disrupting the role of a bank branch network – a core access channel for relationship banking?” This interesting question introduces an important issue: “does bank branch concentration still matter for firms?”.

Advances in digital technology have generated a huge amount of integration in financial markets (Lucey et al., 2018). The advent of digitalization has led banks to resize their branches and increase the use of electronic channels (Nuesch et al., 2015). However, despite digitalization making bank products and services easily accessible at greater distances through online and mobile banking (Khedmatgozar & Shahnazi, 2018; Martins et al., 2014), bank branches have maintained their importance. Some papers have suggested that internet banking performs as a complementary channel relative to traditional bank branch activities rather than as its substitute (De Young et al., 2007; Hernando & Nieto, 2007; Onay & Ozsoz, 2013). However, digitalization, being based on hard quantifiable information, cannot resolve all the asymmetric information problems that arise when SMEs ask for bank loans, because soft information, which is relationship-based information, is difficult to digitalize. In this regard, Ferri and Murro (2015) interestingly pointed out that the financial constraints of informationally opaque firms are greater when loan decisions are based on technology, typically created through hard information. In a similar vein, Berger and Frame (2007) argued that lending decisions based on credit scoring reduce SME access to bank debt.

As a result, the banking business model is moving toward hybrid bank-firm interaction (Nuesch et al., 2015) based on combined digitally and face-to-face acquired information, with the two aspects complementing each other. This implies that digitalization cannot substitute the personal relationships that are formed during physical branch visits, but it could reduce the relevance of bank-firm proximity as entrepreneurs, who are increasingly inclined toward digitalization (Sahut et al., 2021), go to banks less frequently than in the past. Therefore, we expect that digitalization reduces but does not eliminate the relevance of bank branch density in SME use of bank debt. Consequently, we hypothesize:

- Hypothesis 2: Digitalization reduces the positive effect bank-firm physical proximity has on SME debt

## Research design: data, methodology, and variables

### Context of analysis and data

Using the context of Guiso et al. (2004) and others,^6^ we have studied bank-firm physical proximity in the Italian environment, which is a setting in which there are considerable differences in bank branch concentration across different provinces.^7^ Italy is a bank-based economy like many other European countries, such as France, Germany, and Spain. Most Italian banks operate nationwide. In 2019, 76% of the total number of bank branches in Italy belonged to national banks, but cooperative banks (“Banche di Credito Cooperativo” (BCC)) accounted for 18% of total bank branches. Bank debt is the single most commonly used source of financing for SMEs in Italy.^8^ The characteristics of Italian SMEs are very similar to those of most European companies of the same size, as the EU has established a common definition of SMEs among its countries. Hence, this heightens the generalizability of our results. Italy is a relevant case study because of this financial market’s suitability in terms of size, efficiency, and diversity among firms (Cappa et al., 2020a, 2020b). Moreover, in Italy there are important differences in banking development across provinces as well as the north–south divide (Fasano & Deloof, 2021). All these aspects make Italy a considerable setting to investigate our hypotheses.

Our study is based on a large sample of nonfinancial Italian SMEs selected according to the European Commission definition in terms of employees (fewer than 250), annual turnover (less than EUR 50 million), and annual balance sheet total (not exceeding EUR 43 million). The period examined was from 2011 to 2020. We used an unbalanced panel dataset collected from the Orbis database by Bureau van Dijk, which has the most extensive database of financial and business information for SMEs across Europe. We eliminated SMEs operating in financial industries, public administration, education, human health and social work, and creative, arts, and entertainment. We left out economically meaningless observations in terms of input inaccuracies (e.g. non-positive values for total book assets or sales). To limit the potential influence of outliers, we winsorized all the firm-specific variables at the 1st and 99th percentiles (*bank debt, cash holdings, ROA, size, tangibility, intangibles, age, firm growth*). Thus, we obtained a sample of 1,352,926 firm-year observations. Data on the density of bank branches and digitalization in the bank market, per province, came from the Bank of Italy. In particular, it is collected using the website section dedicated to the statistics under the thematic track “Banks, financial institutions, money and financial markets”.^9^ Data on real gross domestic product (GDP) and population per province was collected from the Italian National Institute of Statistics (ISTAT).

### Methodology

We studied the effect bank-firm proximity had on SMEs debt using the two-stage least-squares (2SLS) technique with instrumental variables (IVs) and cluster robust standard errors to account for endogeneity issues.^10^ Furthermore, as robustness econometric tests, we perform the panel fixed effects analysis (in order to eliminate unobservable heterogeneity) with clustered standard errors and the ordinary least squares technique with clustered standard errors^11^ to account for multiple dimensions at the same time as in Cameron et al. (2008).

### Definition of variables

The dependent variable measuring SME financial policies is *bank debt*, which is a proxy for the amount of bank debt used by SMEs. In line with the extant literature (e.g. Rajan & Zingales, 1995), we calculated the level of indebtedness as the ratio of long-term and short-term interest-bearing bank debt scaled by total assets. For the first independent variable, following the approach used by Fasano and Deloof (2021), Guiso et al. (2004), and La Rocca et al. (2010), we measured bank-firm physical proximity by considering the number of national, cooperative, and foreign bank branches scaled to 1000 inhabitants in the province. Prior studies had used this variable because it explains the dimension of bank branch concentration at the local (provincial) level (e.g. Arcuri & Levratto, 2020). As a second independent variable, we calculated *digitalization* as the total number of banking customers using online internet corporate banking services per province divided by the number of inhabitants in the province. It is important to account for internet services demand because bank branches visits of entrepreneurs are less frequent when their firm makes banking operations using online internet services.

We also include a number of firm-specific control variables that may influence the effects we studied. *Cash holdings* is the ratio of cash and cash equivalents scaled by total assets. This variable is important because cash is a substitute for bank debt and, according to pecking order theory, firms with a surplus of cash will use less debt. *ROA* is the ratio of earnings before interest and taxes (EBIT) to total assets and measures profitability. *Size* is the natural logarithm of total assets. More profitable and larger firms typically have easier access to bank debt. *Tangibility* is the ratio of tangible fixed assets scaled to total assets. Tangible assets may increase firms’ financial capacity, since they are used as collateral. *Age* is calculated as the natural logarithm of study year minus year of incorporation. Older firms have a long history that reduces information asymmetries and increases the use of debt. *Firm growth* is calculated as sales in year (*t*) minus sales in year (*t* − 1). Growing SMEs generally require more financial resources. We also control for one provincial characteristic using the variable *GDP growth*, which is measured as growth in real GDP at the provincial level from year (*t* − 1) to year (*t*). *South* is a dummy that equals one if the firm is located in the southern part of Italy and zero otherwise. This variable is important because previous studies in Italy (e.g., Guiso et al., 2004) have shown considerable differences between the northern and southern parts of the country (the so called “north–south divide”). Finally, we included year dummies and industry dummies in our model, as debt policies tend to be industry specific (La Rocca, 2007).

## Empirical results

### Descriptive statistics and correlations

Table 1 shows the descriptive statistics for the variables.

**Table 1:** Descriptive statistics for the sample

	Mean	sd	Min	p25	Median	p75	Max
Bank debt	0.143	0.685	0.000	0.000	0.014	0.241	527.464
Total branch density	0.494	0.170	0.151	0.363	0.487	0.622	1.050
BCC branch density	0.077	0.075	0.000	0.022	0.053	0.118	0.631
Digitalization	0.053	0.018	0.016	0.041	0.053	0.066	0.116
Cash holding	0.141	0.651	0.000	0.013	0.066	0.194	424.428
ROA	0.064	0.128	− 0.453	0.016	0.048	0.104	0.530
Size	6.793	1.529	2.334	5.768	6.778	7.829	10.290
Tangibility	0.248	1.036	0.000	0.045	0.148	0.366	1036.818
Intangibles	0.035	0.121	0.000	0.000	0.004	0.027	43.510
Age	2.454	0.975	0.000	1.792	2.565	3.219	4.956
Firm growth	0.617	3.081	− 0.985	− 0.189	− 0.003	0.291	24.389
GDP growth	0.000	0.021	− 0.175	0.000	0.000	0.000	0.213
South	0.230	0.421	0.000	0.000	0.000	0.000	1.000

**Fig. 1:**
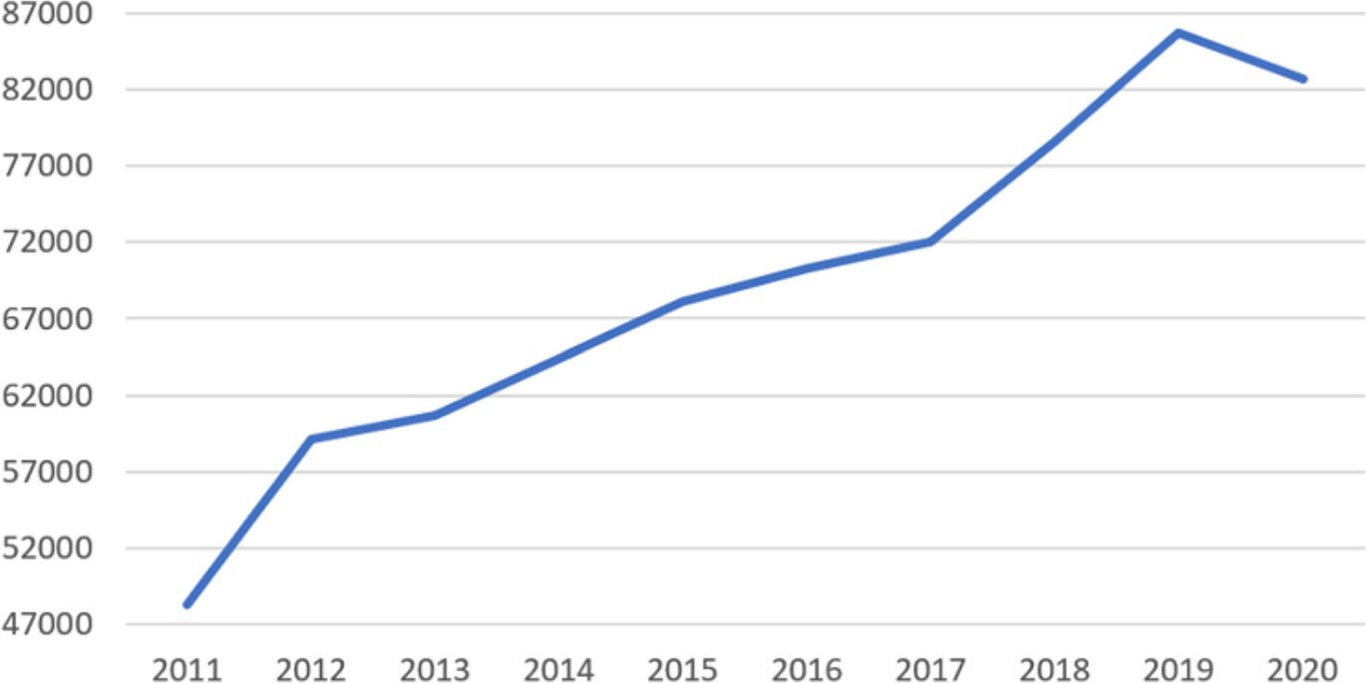
Trend of the mean values of total number of customers using online internet banking services for firms in Italy during the years

These descriptive statistics show that our dependent variable *bank debt* plays a very important role in the financing of SMEs, because, on average, debt constitutes 14.3% of total assets. Moreover, the standard deviation of the variable *bank debt* (0.685) indicates a large degree of variability in the dependent variable across the SMEs in our sample. Table 1 shows that there is also substantial variation with respect to the density of bank branches, while the values for the control variables are in line with existing financial literature contributions. As an additional descriptive statistic, we report the trend of the variable *digitalization* over the sample period in Fig. 1.

Interestingly, this graph shows that during the last decade, the use of internet services increased significantly, except for the year 2020 when firms reduced their activities because of the pandemic.

Table 2 reports the correlation matrix of the variables.

**Table 2:**
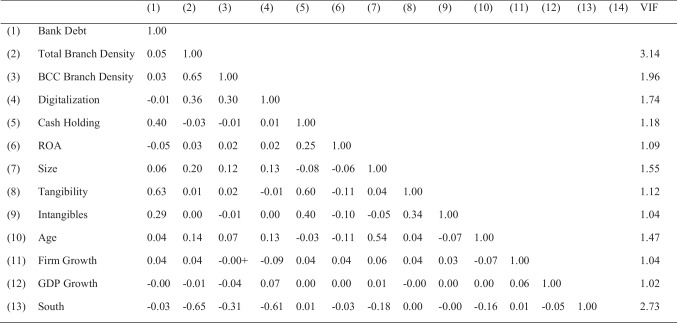
Correlation matrix

Industry dummies are not reported. Correlations different from 0.00 are statistically significant at the 0.01 level

Additionally, we tested possible multicollinearity among the independent variables by using variance inflation factors (VIFs). The maximum VIF in the model is 3.14 (mean of 1.59), which is far below the generally accepted cut-off of 10 (or, more prudently, 5) for regression models (Kutner et al., 2004). Therefore, no bias was detected in the significance of the results.

### Bank-firm proximity, SME financial policies, and the moderating role of digitalization

This section reports the main results of the paper. As previous studies that investigated the role of bank-firm proximity in Italy have settled endogeneity issues by using regressions with IVs, we ran the 2SLS technique using the same IVs as in Fasano and Deloof (2021), Guiso et al. (2004), and La Rocca et al. (2010), who all assessed local banking structures in 1936.^12^

The main analysis results are reported in Table 3.^13^

**Table 3:** Main model: bank-firm physical proximity and SMEs debt, the moderating role of digitalization

Estimation technique:	(1) 2SLS	(2) 2SLS	(3) 2SLS
Dependent variable	Bank debt	Bank debt	Bank debt
Total branch density	0.126^***^ (0.031)	0.126^***^ (0.030)	0.138^***^ (0.032)
Digitalization		− 0.013 (0.010)	0.057^**^ (0.027)
Total branch density			− 0.151^***^ (0.049)
* Digitalization (interaction)			
Cash holdings	− 0.221^***^ (0.018)	− 0.220^***^ (0.018)	− 0.220^***^ (0.018)
ROA	− 0.093^***^ (0.017)	− 0.093^***^ (0.017)	− 0.093^***^ (0.017)
Size	0.017^***^ (0.002)	0.017^***^ (0.002)	0.017^***^ (0.002)
Tangibility	0.081^***^ (0.003)	0.081^***^ (0.003)	0.081^***^ (0.003)
Intangibles	0.107^***^ (0.008)	0.107^***^ (0.008)	0.107^***^ (0.008)
Age	0.006^***^ (0.002)	0.006^***^ (0.002)	0.006^***^ (0.002)
Firm growth	0.000 (0.001)	0.000 (0.001)	0.000 (0.001)
GDP growth	− 0.032 (0.022)	− 0.031 (0.022)	− 0.031 (0.021)
South	0.006 (0.009)	0.005 (0.009)	0.008 (0.010)
Adj. R2	0.031	0.031	0.031
Observations	1,352,926	1,352,926	1,352,926

Industry and year fixed effects are the controls. The* p*-values in parentheses are based on standard errors clustered by provinces and firms. The superscripts denote significance as follows: ^*^p < 0.10, ^**^p < 0.05, ^***^p < 0.01. Standard errors in parentheses

**Fig. 2:**
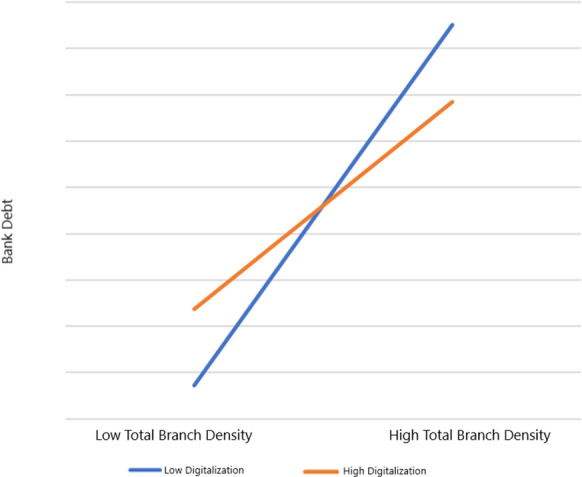
Marginal effect of total branch density on SMEs debt conditioned by digitalization

In column 1, the positive and statistically significant coefficient of the variable *total branch density* reveals that bank-firm proximity considered individually increases SME indebtedness level, confirming our first hypothesis. Column 2 suggests that when the variable *total branch density* and the variable *digitalization* are considered jointly, digitalization seems to have no influence on SME use of debt, but when our regressions include the interaction term (column 3), which is based on the variable *total branch density* multiplied by the variable *digitalization*, we observe that the marginal impact of bank-firm geographical proximity varies according to different levels of digitalization (which now is positive and statistically significant). To better highlight this marginal impact, it is useful to consider a graph that shows the partial effect of the variable *total branch density* on use of debt in SMEs depending on high or low levels of the variable *digitalization*. Thus, to better understand the results, we also provide Fig. 2.^14^

Table 3 (column 3) and Fig. 2 show that the role bank branch concentration plays in SME financial decisions is different in magnitude depending on different levels of digitalization. In particular, the interaction term, which we measured at the 95% confidence interval in regressions, is negative and statistically significant, indicating that the positive effect bank-firm personal contacts have in SME financial policies tends to decrease as the level of digitalization development rises. Therefore, new financial technologies moderate the influence of geographical proximity, and our second hypothesis is confirmed.

In light of these findings, our paper provides two important results. Firstly, it suggests that bank-firm physical closeness is still important, even after the approximately 10 years since the work of La Rocca et al. (2010) and little less than 20 years since the contribution of Guiso et al. (2004). Hence, our analyses based on a recent database confirm the relevance of bank branches proximity. Secondly, our moderation study demonstrates that when firms use more online corporate internet banking, the presence of a close personal bank-firm relationship has a less influence on SMEs using debt. Vice versa, when firms rely less on digital instruments, the density of bank branches in local provincial contexts has a stronger impact on corporate debt choices. In other words, the role of bank-firm human ties is moderated by the level of digitalization. Thus, although digitalization provides hard information for banks, it also reduces the frequency of bank-entrepreneur contacts, having a negative influence on the collection of soft information and, consequently, on the amount of debt in SMEs.

Our evidence suggests that in those contexts where the banking relationship is mostly based on digital interactions, firms’ use of bank debt is lower. This is why an impersonal relationship reduces the availability of soft information that is crucial for loan provision. Therefore, digitalization cannot entirely substitute interaction with human bankers, for which physical proximity still matters. The human ties between a bank and its customers still constitute the core access channel in bank borrowing. Consequently, digitalization supports and accompanies personal banking relationships rather than eliminating them.

### Further tests

#### Cooperative bank branches

By definition, BCCs are local banks, given their legal obligation to operate in limited territorial areas (Stefani et al., 2016). This characteristic puts them geographically closer to SMEs and may make it easier for them to acquire soft information via personal relationships with entrepreneurs, unlike national banks that operate at a greater distance (Bolton et al., 2016; Howorth & Moro, 2006). According to Agarwal and Hauswald (2010), “the requisite soft information is primarily local”, which means BCCs have a competitive advantage in terms of soft information acquisition. BCCs that operate on a much smaller scale probably need digital instruments less when they screen and monitor their customers. Thus, as a further test, Table 4 investigates the effect BCC density has on SME use of debt and the moderating role of digitalization. For the independent variable, we used *BCC branch density*, which is calculated as the number of cooperative branches scaled to 1000 inhabitants in the province. The interaction term is calculated as the variable *BCC branch density* multiplied by the variable *digitalization*. To control for the presence of other bank branches, we introduce the variables *national bank branches* (calculated as the number of national bank branches scaled to 1000 inhabitants in the province) and *foreign bank branches* (the number of foreign bank branches scaled to 1000 inhabitants in the province).

**Table 4:** Further test: BCC Bank branches

Estimation technique:	(1) 2SLS	(2) 2SLS	(3) 2SLS
Dependent variable	Bank debt	Bank debt	Bank debt
BCC branch density	0.086^***^ (0.019)	0.087^***^ (0.019)	0.093^***^ (0.019)
Digitalization		− 0.014 (0.009)	0.038^**^ (0.018)
BCC branch density			− 0.006
* Digitalization (interaction)			(0.011)
National branch density	0.102^***^ (0.018)	0.101^***^ (0.018)	0.106^***^ (0.016)
Foreign branch density	− 0.083 (0.221)	− 0.063 (0.214)	− 0.073 (0.211)
Cash holdings	− 0.221^***^ (0.018)	− 0.221^***^ (0.018)	− 0.221^***^ (0.018)
ROA	− 0.093^***^ (0.017)	− 0.093^***^ (0.017)	− 0.093^***^ (0.017)
Size	0.017^***^ (0.002)	0.017^***^ (0.002)	0.017^***^ (0.002)
Tangibility	0.081^***^ (0.003)	0.081^***^ (0.003)	0.081^***^ (0.003)
Intangibles	0.107^***^ (0.008)	0.107^***^ (0.008)	0.107^***^ (0.008)
Age	0.006^***^ (0.002)	0.006^***^ (0.002)	0.006^***^ (0.002)
Firm growth	0.000 (0.001)	0.000 (0.001)	0.000 (0.001)
GDP growth	− 0.033 (0.023)	− 0.033 (0.023)	− 0.033 (0.023)
South	− 0.002 (0.006)	− 0.003 (0.006)	− 0.002 (0.006)
Adj. R2	0.031	0.031	0.031
Observations	1,352,926	1,352,926	1,352,926

Industry and year fixed effects are the controls. The* p*-values in parentheses are based on standard errors clustered by provinces and firms. The superscripts denote significance as follows: ^*^p < 0.10, ^**^p < 0.05, ^***^p < 0.01. Standard errors in parentheses

Results indicate that when we consider cooperative banks branches, digitalization does not moderate the relationship between bank-firm proximity and debt. This is interesting, but not surprising. Indeed, these findings confirm the expectation that cooperative banks will have a robust, especially close relationship with local entrepreneurs. The table also shows that *national bank branches* positively affects SMEs’ use of debt, while *foreign bank branches* does not affect such a relationship.

#### The role of the COVID-19 crisis

**Fig. 3:**
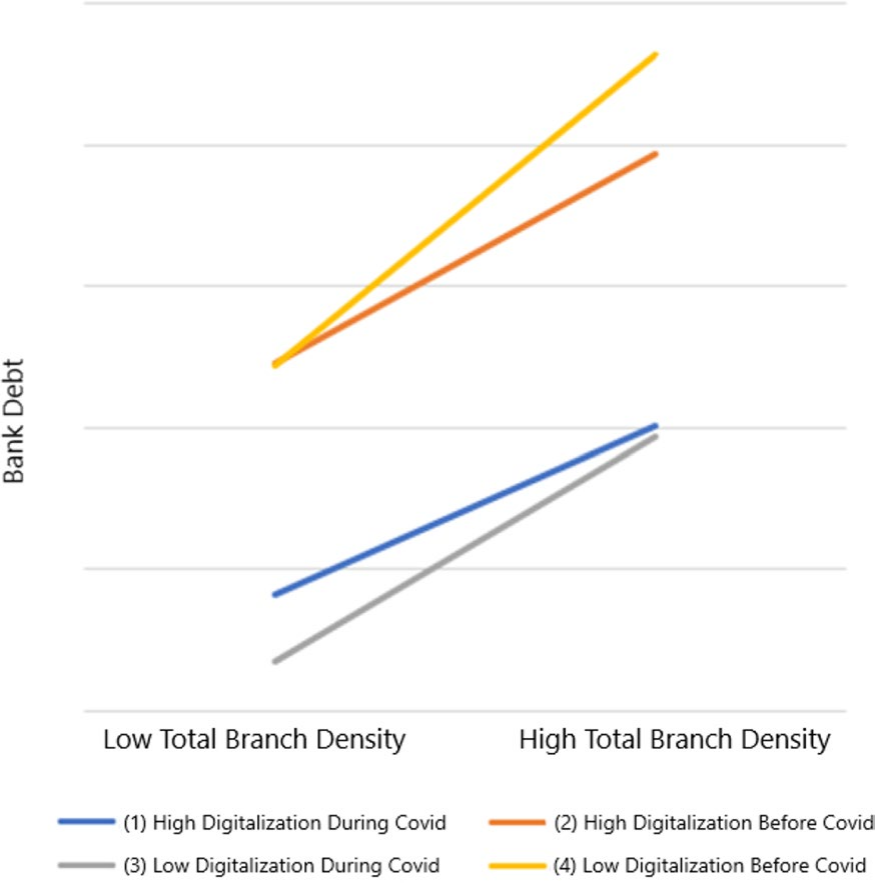
Marginal effect of total branch density on SMEs debt conditioned by digitalization and COVID-19 crisis

The 2020 COVID-19 crisis stimulated emerging literature on the impact the pandemic has had on corporate financing decisions (e.g. Fahlenbrach et al., 2021; Vo et al., 2021), especially for SMEs (Belitski et al., 2022; Fasano et al., 2022). Our second further test provides new evidence for this stream of research. In particular, we studied the effect of the COVID-19 crisis using three-way interaction regressions, where the relationship between bank-firm human proximity and firms’ use of debt is moderated by the variable *digitalization* and the variable *dummy COVID-*19, which is equal to one if the year is 2020 and zero otherwise. Thus, we ran a regression analysis, including all three independent variables, all three pairs of two-way interaction terms, and the three-way interaction term. Table 5 and Fig. 3 show the regression results.

**Table 5:** Further and robustness tests

Estimation technique:	(1) Panel FE Further test COVID-19 effect	(2) Panel FE Further test small and large SMEs	(3) Panel FE Robustness test (alternative digitalization measure)	(4) Panel FE	(5) OLS cluster
Dependent variable	Bank debt	Bank debt	Bank debt	Bank debt	Bank debt
Total branch density	0.111^***^ (0.020)	0.096^***^ (0.032)	0.177^***^ (0.010)	0.112^***^ (0.020)	0.102^***^ (0.002)
Digitalization	0.029^**^ (0.013)	0.022^*^ (0.013)		0.029^**^ (0.013)	0.038^***^ (0.012)
Dummy COVID-19	− 0.046^**^ (0.022)				
Dummy small SMEs		− 0.029 (0.017)			
Total branch density	− 0.076^***^ (0.011)	− 0.059^***^ (0.010)		− 0.076^***^ (0.008)	− 0.113*** (0.009)
* Digitalization (interaction)					
Total branch density	− 0.049 (0.016)				
* Dummy COVID-19 (interaction)					
Digitalization * dummy COVID-19 (interaction)	0.010 (0.027)				
Total branch density	0.057 (0.058)				
* Digitalization * dummy COVID-19 (interaction)					
Total branch density * Dummy small		0.032 (0.025)			
SMEs (interaction)					
Digitalization * dummy		0.014^**^			
Small SMEs (interaction)		(0.006)			
Total branch density		− 0.036^**^ (0.014)			
* Digitalization * dummy small SMEs (interaction)					
Digitalization 2			0.041^***^ (0.005)		
Total branch density * Digitalization 2 (interaction)			− 0.077^***^ (0.005)		
Cash holdings	0.044 (0.016)	− 0.046^***^ (0.016)	0.044 (0.072)	− 0.045^***^ (0.002)	− 0.199^***^ (0.010)
ROA	− 0.121^***^ (0.009)	− 0.121^***^ (0.009)	− 0.121^***^ (0.009)	− 0.122^***^ (0.010)	− 0.095^***^ (0.013)
Size	0.021 (0.024)	0.009 (0.027)	0.012 (0.024)	0.012 (0.024)	0.017^***^ (0.002)
Tangibility	0.370^***^ (0.071)	0.119^***^ (0.005)	0.118^***^ (0.004)	0.119^***^ (0.005)	0.098^***^ (0.008)
Intangibles	0.057^**^ (0.107)	0.057^***^ (0.011)	0.058^**^ (0.105)	0.058^***^ (0.011)	0.123^***^ (0.010)
Age	0.025^*^ (0.015)	0.028 (0.016)	0.025^*^ (0.015)	0.026^*^ (0.015)	0.005^**^ (0.002)
Firm growth	− 0.000 (0.001)	0.001 (0.001)	− 0.000 (0.001)	0.001 (0.001)	0.000 (0.001)
GDP growth	− 0.033 (0.014)	− 0.033^**^ (0.014)	0.004 (0.100)	− 0.033^**^ (0.014)	− 0.043^***^ (0.010)
South	− 0.001 (0.001)	− 0.001 (0.001)	− 0.003^***^ (0.001)	0.029 (0.001)	− 0.001 (0.005)
Adj. R2	0.009	0.009	0.009	0.008	0.032
Observations	1,352,926	1,352,926	1,352,926	1,352,926	1,352,926

Industry and year fixed effects are the controls. The* p*-values in parentheses are based on standard errors clustered by provinces and firms. The superscripts denote significance as follows: ^*^p < 0.10, ^**^p < 0.05, ^***^p < 0.01. Standard errors in parentheses

Interestingly, our results suggest that the coronavirus pandemic decreased the overall amount of bank debt used by SMEs, but as expected, it did not change the magnitude of the reduction effect the variable *digitalization* had on the relationship between bank-firm physical proximity and SME use of debt. Future studies could deepen these findings also studying the behaviour of SMEs during the year 2021.

#### Small versus large SMEs

**Fig. 4:**
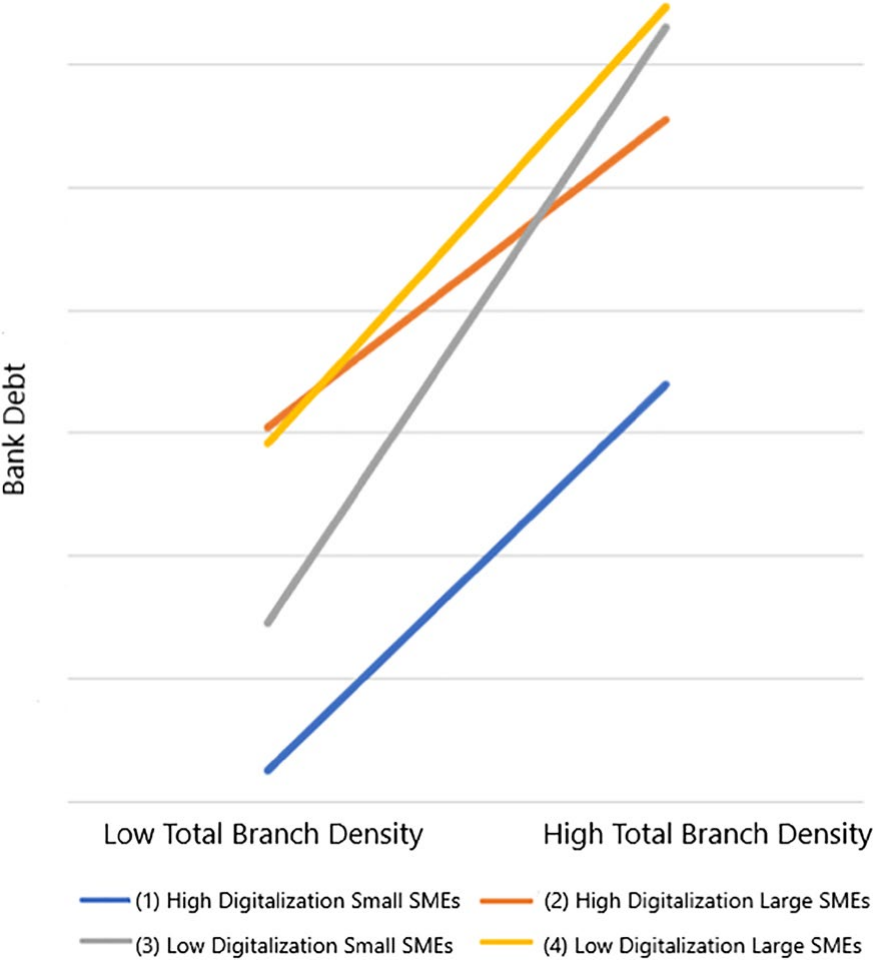
Marginal effect of total branch density on SMEs debt conditioned by digitalization considering large and small SMEs

Table 5 also reports our results considering firms that are differently sensitive to asymmetric information problems. More specifically, we used firm size as a proxy for asymmetric information (Bigelli & Sánchez-Vidal, 2012), and we considered subsamples of small and large SMEs based on the median value of the variable *size*. We investigated the role of corporate dimension using three-way interaction regressions, where the relationship between the variables *total branch density* and *bank debt* is moderated by the variable *digitalization* and the variable *dummy small*, which is one when the variable *size* is lower than the median value and zero otherwise. Thus, we study the marginal impact of *digitalization* depending on the firm’s dimension. First, we found that the amount of bank debt used is higher for large SMEs. Second, we observed, in line with the main results, that for both large and small SMEs, low levels of the moderating variable *digitalization* are related to a slightly stronger effect of bank branch concentration on debt. However, when digitalization is low, the positive effect of bank branch concentration on SME use of debt seems to be stronger for smaller SMEs. Indeed, as expected, SMEs are more informationally opaque and, as such, benefit most from bank-firm physical proximity. This result is not surprising, because the strong bargaining power large SMEs have during debt contract negotiations, thanks to higher levels of variables such *tangibility* or *age*, makes the level of digitalization development less relevant for their financial choices compared to small SMEs. This finding can be also seen in Fig. 4.

### Robustness tests

#### Alternative digitalization measure

For our first robustness test, we used an alternative proxy for the variable measuring digitalization. Specifically, considering the expansion of mobile banking (Konte & Tetteh, 2023; Picoto & Pinto, 2021), we calculated the variable *digitalization 2* as the total number of mobile banking services used by bank customers per province scaled to 1000 inhabitants in the province. The regression results reported in Table 5, column 3, confirm the validity of our findings, using the alternative measure of digitalization as well.

#### Panel fixed effects and OLS cluster techniques using clustered standard errors

Before launching our panel regressions, we first ran the Hausman test, which suggests that the fixed effects model better fits our data. Additionally, we ran a parm test which suggests that time fixed effects are needed. Then, we performed the OLS cluster technique. The latter approach is important because it makes it possible to control for observations that are correlated under two dimensions (province and firm-level). OLS cluster regressions correct standard errors regarding the possible dependence of the residuals within clusters. The results of the Panel FE and OLS regressions confirm our findings, as shown in Table 5, columns 4 and 5, respectively.

#### Placebo test

As an additional robustness test, we applied a placebo test. The sample has a very high number of observations which could affect the statistical significance of the findings (Athey & Imbens, 2017). To avoid having this number lead to false statistically significant results, we ran a placebo test in which we randomly assigned the variable *branch density* to each firm in our sample 200 times, and each time, we re-estimated the regression using the re-shuffled independent variable *bank debt*. We expected that in this setting, the variable *branch density* would not significantly affect SME use of debt. When we ran the placebo test 200 times, we found that the estimated coefficients of the variable *branch density* were not statistically significant at the 10% level in more than 90% of cases.^15^ Therefore, the results of the tests confirmed the robustness of our findings, which are consequently not influenced by chance.

#### Other robustness tests

For some final robustness tests, we launched our regressions first without winsorizing the firm-specific variables, then we account for debt maturity (Ahangar, 2021) by making the dependent variable the ratio between long-term and short-term interest-bearing bank debt scaled to long-term and short-term interest-bearing bank debt plus equity, and finally by making the dependent variable the natural logarithm of the variable *bank debt*. Moreover, we control for the number of companies per province that could affect the demand for digital services. We ran our main model regressions adding the new variable *n_companies* (that is calculated as the number of companies per province scaled to the number of inhabitants in the province with refers to the year 2017, which is a year in the middle of our panel sample). All the results showed no significant differences with respect to our main model.

## Conclusion and implications

Digitalization is one of the technologies that is transforming the banking sector, and it has received a great deal of attention from scholars and practitioners all over the world. Digitalization allows banks to provide services more efficiently than in the past by acquiring a huge amount of additional information about firms. This revolution integrates the work of human bankers in the process of mitigating information asymmetry problems, such as adverse selection and moral hazard problems. In this framework, based on asymmetric information theory, our paper has scrutinized whether and to what extent digitalization moderates the effect that bank-firm geographical proximity has on the amount of debt used by Italian SMEs.

Our findings, supported by a number of robustness tests, suggest that the positive effect produced by physical closeness between a bank and a firm will decrease as the level of digitalization rises, which indicates that new financial technologies will moderate the positive value of bank branch concentration. It seems that when the banking relationship is predominantly based on digital channels and bank-entrepreneur personal interactions are low, firms use a smaller amount of bank debt. The explanation for these findings can be found in the asymmetric information perspective. Indeed, when the banking relationship is face to face, the availability of soft information is higher, and firms can obtain more credit. Vice versa, when there is a high level of digital and personal contacts are fewer, there is less soft information available, and firms use less bank debt. Moreover, it is interesting that the moderating effect observed does not occur in the presence of cooperative bank branches that operate in local contexts and for which soft information is available independently of the level of digitalization. Moreover, we also found that the recent COVID-19 crisis reduced the amount of bank debt used by SMEs, but it did not influence the magnitude of the moderating effect of digitalization. In other words, the role of information asymmetries did not change during the pandemic. Finally, we found that when the level of digitalization is low, the positive influence that bank-firm closeness has on SMEs using debt is stronger for smaller SMEs, where human relationships are more important than for large SMEs, when it comes to the information collection process. Therefore, the latter findings also confirm the importance of information asymmetries, especially for more informationally opaque firms.

Our work also has important implications for managers, financial institutions, and governments. These findings indicate that managers should recognize that bank branch proximity is still important, even in a digital environment. Indeed, while internet banking provides standard quantifiable information about borrowers, human interactions allow banks to acquire “soft” qualitative information that is at the heart of the decision-making process. Therefore, despite the fact digitalization is changing the bank-customer relationship, it is unlikely that digital technologies will replace personal contacts in the long run. On the contrary, it is likely that digital and direct personal connections will coexist. But how do digitalization and personal relationships interact with each other? Human bankers adopt their discretional decisions on the basis of both soft information acquired through personal contacts and quantitative information provided by digitalization. We therefore suggest a theoretical perspective that supports the complementarity of the physical and digital worlds. A recent study in the literature has examined the impact of so-called “phygital transformation” on firms (e.g. Cennamo et al., 2022), and we aim to contribute to and stimulate this emerging phenomenon. However, digitalization could take huge steps forward. A further advance could be the development of artificial intelligence techniques to better support loan officers’ strategic and qualitative decisions. Technological research could lead to the use of digitalization to exploit machine learning techniques for applications that guide banks not only toward choices based on quantitative data, but that also provide support for strategic/qualitative decisions, with a consequently strong positive impact on bank-firm relationships. This will create a unique new banking business model where digitalization constitutes an opportunity to reduce the discretion of decisions based on “soft” information. This will also reduce errors in loan assessment and, consequently, financial constraints. Financial institutions and governments should take into considerations such aspects when developing new policies in the banking industry. The hope is to exploit digitalization further to help bankers make decisions without abolishing the personal interface that is at the heart of the banking relationship. Consequently, it is paramount to balance digital adoption with human experiences in banking.

We also provide useful implications for policymakers. As the growth of digitalization has generated turbulence in banking markets, governments and central banks should consider this trend carefully and implement proper regulations that make digitalization an opportunity rather than a threat. Governments should thus regulate digital development, steering it in the right direction. In particular, digitalization should be used to reduce asymmetric information problems, and bankers should be careful when the use of hard information could be an obstacle to loan provision. It is precisely when hard information suggests it is not advisable to grant a loan that close personal ties should be considered important. It is thus imperative to remember that digitalization does not substitute banks when it comes to their most important key functions, because close relationships in banking are still essential and the significance of geographic distance is still crucial in lending decisions.

A possible limitation of our study is that it focuses on a single country context. Despite SMEs’ definition is the same for all the European countries, national features could affect the relationships studied (e.g. internet adoption depends on national culture, as pointed out by Blagoev & Shustova, 2019). However, our results could be partially generalized to those contexts that have bank-based and civil law systems as in Italy. In light of this, a new direction of research could investigate the game-changing role of banking digitalization in different countries.

## Data Availability

The data that support the findings of this study are available from third party: BvD. Restrictions apply to the availability of these data, which have been used under license for this study, and we not allowed to publicly depositing the data. However, we can share the stata commands and arrange a phone call or something similar with the editor to show how we got our results opening the raw data and then running the stata commands to get descriptive and regressions results.

## Footnotes

1 “Fintech is a new financial industry that applies technology to improve financial activities” (Schueffel, 2016).

2 Banking relationship is intended as “the connection between a bank and customer that goes beyond the execution of simple, anonymous, financial transactions” (Ongena and Smith,^ 2000^).

3 Information asymmetry is defined as “the difference in information between the managers of the firm and the market” (Dierken, 1991) and is particularly important in the banking context (Liao et al., 2009).

4 According to Petersen (2004) “soft” information “is difficult to completely summarize in a numeric score”, while “hard information is quantitative, easy to store and transmit in impersonal ways, and its content is independent of the collection process”.

5 Big data is large datasets that have the ability to generate valuable information and insights (Cappa et al., 2021);(Johnson et al., 2017).

6 See, for example, La Rocca et al. (2010), Deloof and La Rocca (2015), and Fasano and Deloof (2021).

7 In 2022 Italy accounts for 107 provinces corresponding to the NUTS3 areas in the EU classification according to the statistical office of the European Union (Eurostat dataset).

8 Source: Bank of Italy, Annual Report 2020 available at https://www.bancaditalia.it/pubblicazioni/relazione-annuale/2020/rel_2020.pdf

9 Available at https://www.bancaditalia.it/statistiche/index.html

10 In line with prior studies in this field of research based on Italy (e.g. Fasano & Deloof, 2021; Guiso et al., 2004).

11 We followed the approach of Deloof and La Rocca (2015) using the Mitchell Petersen’s Stata routine to cluster standard errors with two dimensions (available at https://www.kellogg.northwestern.edu/faculty/petersen/htm/papers/se/se_programming.htm).

12 These structures were established by factors unrelated to local economic development. The legislation introduced in Italy in 1936 limited the growth of the national banking sector, and consequently, many of the provincial differences present in 1936 still exist today. The instruments consider the quantity of bank branches in 1936, the number of different banks, the number of “popular bank” branches, and 1936 branch density in the province of the SMEs involved. “Popular banks” are larger cooperative banks.

13 Before performing our regressions, we scaled the variable digitalization by 1000 to improve the reading and the intelligibility of the regression coefficients.

14 Figure 1 is based on the Jeremy-Dawson graph. For further information, see www.jeremydawson.co.uk/slopes.htm.

15 Results of the placebo tests are available upon request to the authors.
